# α-Synuclein-derived lipoparticles in the study of α-Synuclein amyloid fibril formation

**DOI:** 10.1016/j.chemphyslip.2019.02.009

**Published:** 2019-05

**Authors:** Marcel Falke, Julian Victor, Michael M. Wördehoff, Alessia Peduzzo, Tao Zhang, Gunnar F. Schröder, Alexander K. Buell, Wolfgang Hoyer, Manuel Etzkorn

**Affiliations:** a Institut für Physikalische Biologie, Heinrich-Heine-University Düsseldorf, Germany; b Institute of Complex Systems (ICS-6), Forschungszentrum Jülich, Germany

**Keywords:** α-Synuclein, Membrane interaction, Nanodiscs, αSyn-LiPs, Amyloid formation, Lipid-modulated protein aggregation

## Abstract

Aggregation of the protein α-Synuclein (αSyn) is of great interest due to its involvement in the pathology of Parkinson’s disease. However, under in vitro conditions αSyn is very soluble and kinetically stable for extended time periods. As a result, most αSyn aggregation assays rely on conditions that artificially induce or enhance aggregation, often by introducing rather non-native conditions. It has been shown that αSyn interacts with membranes and conditions have been identified in which membranes can promote as well as inhibit αSyn aggregation. It has also been shown that αSyn has the intrinsic capability to assemble lipid-protein-particles, in a similar way as apolipoproteins can form lipid-bilayer nanodiscs. Here we show that these αSyn-lipid particles (αSyn-LiPs) can also effectively induce, accelerate or inhibit αSyn aggregation, depending on the applied conditions. αSyn-LiPs therefore provide a general platform and additional tool, complementary to other setups, to study various aspects of αSyn amyloid fibril formation.

## 1 Introduction

The aggregation of the protein α-Synuclein (αSyn) into amyloid fibrils is associated with the pathology of Parkinson’s disease (Spillantini et al., 1997; Luk et al., 2012; Jucker and Walker, 2013; Tuttle et al., 2016; Butterfield and Lashuel, 2010; Auluck et al., 2010). Notably, purified αSyn on its own is kinetically stable in its intrinsically disordered, monomeric form, even at high concentrations and/or temperatures. Therefore, to study the process of αSyn amyloid fibril formation, experimental conditions are typically chosen under which aggregation is promoted. One frequently applied αSyn aggregation assay setup uses for example a glass bead inside the sample solution in combination with agitation of the sample (Giehm and Otzen, 2010; Wordehoff and Hoyer, 2018) resulting in strongly enhanced aggregation. Factors which may promote αSyn aggregation in this ‘glass-bead assay’ (GB-assay) include (i) frequent scission of fibrils, constantly increasing the number of fibril ends available for elongation (Shvadchak et al., 2015), and (ii) increased detachment of αSyn aggregates from the air-water-interface, where fibril nuclei preferentially form (Campioni et al., 2014; Pronchik et al., 2010). GB-assays have been used/optimized by numerous groups and in many cases lead to improved reproducibility in the aggregation behavior, rendering them useful for the characterization of factors that for example interfere with αSyn aggregation (Giehm and Otzen, 2010; Chorell et al., 2015; Huang et al., 2006; Wordehoff et al., 2017; Yoshimura et al., 2017). On the other hand, the intrinsic properties of the GB-assay can mask key processes of amyloid fibril formation. For example, the highly-induced fragmentation rate renders it difficult to detect other secondary processes, such as secondary nucleation on the fibril surface (Buell et al., 2014; Gaspar et al., 2017). Furthermore, the primary nucleation at the air-water interface impairs the quantification of the effects of other surfaces, such as liposomes (Galvagnion et al., 2015) or nanoparticles (Vacha et al., 2014) on the nucleation rate.

While its native function is not yet fully understood, αSyn is known to interact with membranes and a physiological role of αSyn in membrane-associated processes has been proposed (Gitler et al., 2008; Bellani et al., 2010; Diao et al., 2013; Fusco et al., 2016;). It has also been shown that the presence of lipids can modulate αSyn aggregation behavior (Zhu et al., 2003; Dikiy and Eliezer, 2012). Using small unilamellar vesicles (SUVs) formed with anionic lipids, conditions have been identified that can enhance aSyn aggregation, providing a useful alternative to GB-assays (Galvagnion et al., 2015). We have recently shown that similar results can be obtained using lipid-bilayer nanodiscs formed with anionic lipids and the membrane scaffold protein MSP1D1 (Viennet et al., 2018). While both membrane mimetics can modulate αSyn aggregation in a similar manner, it appears that the presence of nanodiscs leads to the formation of fibrils with a morphology very similar to the ones formed in the absence of lipids (Viennet et al., 2018), i.e. mature fibrils with diameters in the range of 8–10 nm. SUV-induced aggregation, on the other hand, leads to morphologically distinct short fibrils (Galvagnion et al., 2015), which have been shown to convert into mature fibrils after heating to above 50 °C (Brown et al., 2018). The application of nanodiscs and SUVs may therefore provide complementary information useful to disentangle the different processes involved in lipid-induced αSyn aggregation.

Interestingly, it has also been shown that αSyn, due to its amphipathic character, can stabilize lipid bilayers analogous to the membrane scaffold protein (MSP) (Mizuno et al., 2012; Varkey et al., 2013) and that stable αSyn-lipid particles (αSyn-LiPs) can be assembled in vitro using a similar approach as for nanodisc preparations (Eichmann et al., 2016, 2017). While the occurrence and possible physiological role of αSyn-LiPs is unclear, they may display useful features that could be exploited for in vitro studies.

Here we investigate the behavior of these αSyn-LiPs in αSyn aggregation assays. In line with results obtained on nanodiscs, we show that depending on the ratio of ‘free’ αSyn to αSyn-LiPs, the presence of the lipid particles can either inhibit or accelerate αSyn aggregation. In comparison to SUVs, αSyn-LiPs appear to be more stable, simplifying their handling. Additionally, the presence of αSyn-LiPs, in contrast to SUVs (Brown et al., 2018), does not noticeably alter fibril morphology and does not lead to kinetically trapped fibrils. In comparison to MSP1D1-derived nanodiscs, usage of αSyn-LiPs reduces the aggregation setup to a two-component system, simplifying sample preparation and eliminating potential effects of the membrane scaffold protein. Our results suggest that αSyn-LiPs may therefore be a useful complementary tool to study different aspects of lipid-induced αSyn aggregation.

## 2 MaterialS and methods

### 2.1 αSyn and N-terminally acetylated αSyn expression and purification

αSyn in the pT7-7 vector was expressed in *E. coli* BL21 DE3. For acetylated αSyn, the N-terminal acetylation enzyme NatB from *Schizosaccharomyces pombe* was coexpressed in a second vector, pNatB (Johnson et al., 2010). Expression was conducted in 50 mM phosphate-buffered 2YT-medium (pH 7.2) with 0.4% glycerol and 2 mM MgCl_2_, protein production was induced at OD 1–1.2 with 1 mM IPTG and ran for 4 h at 37 °C.

Purification of acetylated and non-acetylated α-syn was carried out as previously described^8^, some changes to the protocol have been made. A cell pellet of 1 l culture was dissolved in 20 ml of 50 mM Tris-HCl pH 8, 150 mM NaCl, 5 mM EDTA containing a protease inhibitor tablet (cOmplete Mini, Roche) and cells were lysed by sonication with a MS72 tip connected to a Bandelin Sonopuls sonicator (30% Amplitude, 1.5 s ON, 3.5 s OFF, 5 min) on ice. Cell debris was pelleted at 15,000∙*g* for 20 min at 4 °C. The supernatant was boiled at 95 °C for 15 min to precipitate unwanted proteins which were pelleted at 15,000∙*g* for 20 min and 4 °C. After that, the supernatant was sterile-filtered and αSyn was precipitated by gradually adding 4 M ammonium sulfate solution until a concentration of 1.75 M was reached. αSyn was pelleted at 15,000∙*g* for 20 min at 4 °C, the pellet was then dissolved in 10 ml of 50 mM Tris-HCl pH 8 and dialysed against 1.8 l of 50 mM Tris-HCl pH 8 overnight at 4 °C. Subsequently, αSyn was loaded onto a 5 ml HiTrap Q HP anion exchange column (GE Healthcare). Impurities were eluted by washing the column with 8 M Urea, 5 mM Dithiothreitol in 50 mM Tris-HCl pH 8, 100 mM NaCl for 30 min. αSyn eluted at around 250–300 mM NaCl in a 20-column volume gradient from 100 to 500 mM NaCl in 50 mM Tris−HCl pH 8. αSyn was then again precipitated with ammonium sulfate as described above, dissolved in an appropriate volume of 25 mM potassium phosphate buffer pH 7.4 and dialysed extensively against 1.8 l of the same buffer overnight at 4 °C. αSyn concentration was determined by measuring UV absorption at 275 nm and using an extinction coefficient of 5600 M^−1^ cm^−1^.

### 2.2 αSyn-LiP assembly

αSyn-LiPs were assembled according to established protocols (Eichmann et al., 2016). In short, POPG or POPC lipids (Avanti) were suspended in lipid resuspension buffer (20 mM Tris-HCl pH 7.5, 100 mM NaCl, 60 mM Na-cholate, 5 mM EDTA) to a final concentration of 26 mM. Monomeric αSyn and lipids were mixed at a molar ratio of 1:40. 20% w/v of previously washed Biobeads SM-2 (Biorad) were added and the mixture was incubated at room temperature overnight. The Biobeads were removed by centrifugation and once again 20% w/v were added for an additional 4 h. Finally, αSyn-LiPs were purified by SEC on a HiLoad 16/600 Superdex 200 pg column or analyzed using a 10/300 Superdex 200 column (GE Healthcare) equilibrated with 20 mM sodium phosphate pH 7.4. NaCl concentrations of 50 mM (low salt), 150 mM (medium salt) or 300 mM (high salt) were used at a flow rate of 1 ml min^–1^ on an ÄKTA Pure FPLC (GE Healthcare). αSyn-LiPs were concentrated to the desired molarity using a Vivaspin concentrator with a 10 kDa MWCO. Where provided αSyn-LiP concentrations are calculated based on the αSyn absorbance measurements and the assumption of 8 αSyn molecules per αSyn-LiP.

### 2.3 MSP1D1-nanodiscs preparation

Expression and purification of MSP1D1 as well as nanodisc assembly was carried out as reported before (Viennet et al., 2018). 100% POPG lipids and MSP1D1 after proteolytic cleavage of the Histidine tag were used for all MSP1D1 nanodiscs used in this study.

### 2.4 Thioflavin T (ThT) fluorescence aggregation assays

#### 2.4.1 Influence of αSyn-LiPs on lipid-independent αSyn fibril formation (GB-asssay)

In order to study the influence of αSyn-LiPs on αSyn fibril formation, experimental conditions were chosen such that αSyn fibril formation occurs spontaneously by interface-driven nucleation and amplifies through fibril fragmentation. 25 μM of acetylated αSyn were mixed with αSyn-LiPs at molar ratios of 8:1 (3.125 μM αSyn-LiPs), 16:1, 64:1, and 128:1 in 20 mM potassium phosphate buffer pH 7.4 with 50 mM KCl, 0.05% NaN_3_ and 10 μM Thioflavin T (ThT). Duplicates of 80 μl each were pipetted into half area 96-well plates with non-binding surface (Corning No. 3881, black, clear bottom) containing a glass bead (2.85–3.45 mm diameter, Carl Roth) for mixing and incubated at 37 °C for 5 days. Thioflavin T fluorescence was excited at 445 nm and measured at 485 nm every 20 min with 15 s of orbital shaking at 180 rpm prior to the measurement in a plate reader (Tecan Spark 10 M). Note that in order to provide a most accurate comparison between MSP1D1 ND and αSyn-LiPs, both were prepared in parallel under identical conditions, including assembly and SEC purification at NaCl concentrations of 150 mM (medium salt).

#### 2.4.2 Nucleation-sensitive assays

We have previously reported that the presence of nanodiscs can accelerate nucleation of αSyn amyloid fibrils under conditions that minimize the intrinsic nucleation rate (Viennet et al., 2018). A similar setup, i.e. quiescent conditions and protein-repellent plate surfaces, was used to determine possible effects of αSyn-LiPs on the nucleation rate of αSyn. 25 μM (final concentration) of acetylated αSyn was mixed with αSyn-LiPs at molar ratios of 4:1, 8:1, 16:1, 32:1, 64:1, 128:1, 256:1, 512:1, and 1024:1. Assays were performed in 20 mM sodium phosphate buffer pH 7.4 with 50 mM NaCl, 0.05% NaN_3_ and 10 μM Thioflavin T (ThT). Multiples of 30 μl were pipetted into 384-well plates with non-binding surfaces (Greiner 71900, black, non-binding). The samples were incubated at 37 °C in a plate reader (Tecan Spark 10 M or Tecan infinite M1000PRO) for up to 17 days during which aggregation was monitored by exciting ThT fluorescence at 445 nm and measuring emission at 485 nm every 20 min.

### 2.5 Microfluidics measurements

A Fluidity One instrument (Fluidic Analytics Ltd., Cambridge, U.K.) was used for microfluidic diffusional sizing measurements (Arosio et al., 2016) with post separation labeling (Yates et al., 2015) using injection moulded disposable plastic chips. Triplicate measurements for each condition were performed and average hydrodynamic radii with standard deviation error margins are plotted. αSyn-LiPs concentrations were in the range of 1 μM.

### 2.6 Dynamic light scattering (DLS)

**Fig. 1:**
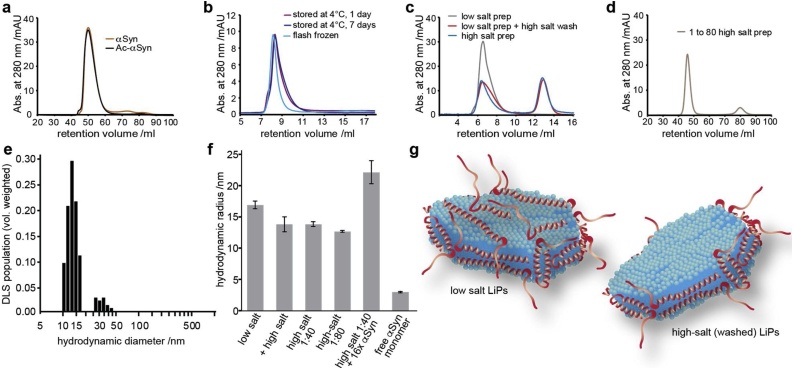
**Preparation and characterization of αSyn-LiPs**. a–d) SEC analysis of different αSyn-LiP preparations. a) αSyn-LiPs formed with acetylated (black) and non-acetylated (orange) αSyn (preparative scale). b) Analytical SEC of acetylated αSyn-LiPs after storage for one day at 4 °C (purple) or one week at 4 °C (dark blue) or after flash freezing with liquid nitrogen and storage at −20 °C (light blue). c) αSyn-LiP preparation in low-salt conditions (grey). Same sample but after incubating and running in high-salt buffer (red) as well as αSyn-LiPs directly assembled in high-salt buffer (blue). d) αSyn-LiPs assembled in high-salt conditions with 2-fold more lipids per αSyn (i.e. molar ratio 1:80 - αSyn:POPG). e) Histogram of αSyn-LiP particle sizes as determined via dynamic light scattering (DLS). f) Measured hydrodynamic radii of indicated samples using a microfluidic setup (see text for more details).g) Possible model of αSyn-LiPs assembled with anionic lipids and either low-salt (left) or high-salt (right) conditions. Note that αSyn orientation at membrane edges is unknown.

DLS was performed on a submicron particle sizer, Nicomp 380 (Particle Sizing Systems Nicomp, Santa Barbara, CA). Data were analyzed with the Nicomp algorithm using the volume-weighted Nicomp distribution analysis. Additional data analysis is shown in supplementary Fig. 2. POPG αSyn-LiPs prepared under low salt conditions, directly after SEC elution were measured. Note that analysis shown in Fig. 1e identifies also a species of particle sizes > 500 nm with a (volume) contribution of 0.03% that is not visible in the graph.

**Fig. 2:**
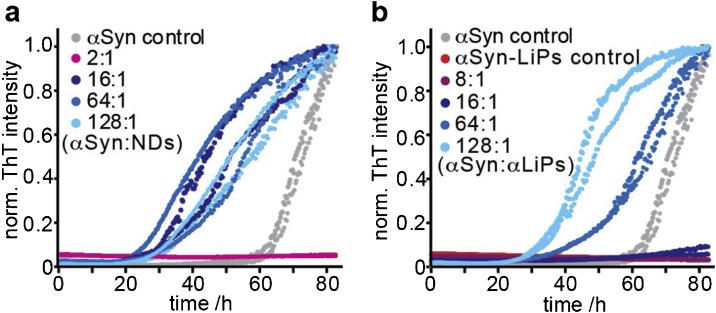
**aSyn-LiPs in direct comparison to classical MSP-derived nanodiscs as a tool in aggregation assays.** Comparison between MSP1D1 NDs (a) and αSyn-LiPs (b) in a GB-aggregation assay. αSyn aggregation kinetics, as measured by increase in ThT fluorescence, in the absence of NDs/LiPs (grey) and in the presence of indicated ratios of monomeric αSyn per NDs (a) or per αSyn-LiP (b) are shown. Note that monomeric αSyn concentration is kept constant and αSyn-LiP particle concentration was estimated assuming an average of 8 αSyn proteins per LiP as reported before (Eichmann et al., 2016). Duplicate measurements are shown with same color. Both the ND and the αSyn-LiP system are able to inhibit as well as to accelerate αSyn aggregation as compared to αSyn in the absence of lipid particles (grey). However, the ratios of added αSyn monomers per lipid particle that lead to inhibiting or accelerating behavior differ between αSyn-LiPs and NDs.

### 2.7 Fourier transform infrared spectroscopy (FITR)

Infrared spectroscopy using the Direct Detect® system (EMD Millipore) was used to quantitatively determine the concentration of protein and lipids in the LiPs. The instrument uses a calibration via a BSA standard (Sigma) to quantify the protein abundance at multiple wavenumbers, including 1650 cm^−1^. POPG lipid signal was calibrated manually using several dilutions of POPG in Na-cholate buffer. Signal from the C-H symmetric stretching vibrational populations between 2870 and 2840 cm^−1^ was used to quantify lipid signals (see supplementary Fig. S1 for data and more information).

### 2.8 Electron microscopy (EM)

**Fig. 3:**
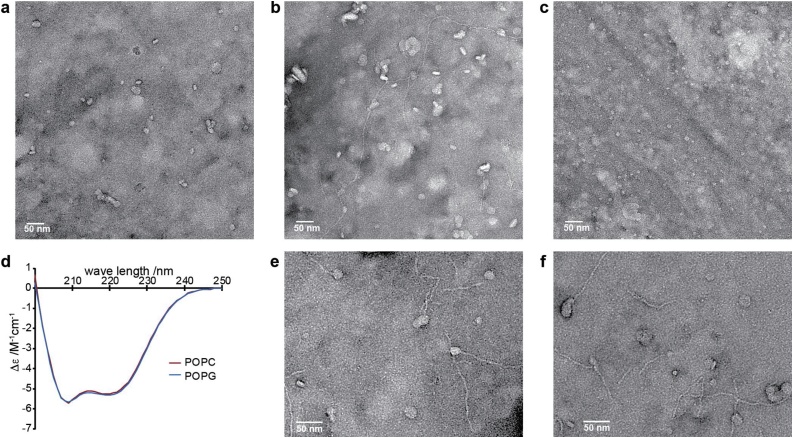
**αSyn-LiPs properties after SEC elution.** a) EM image of POPG αSyn-LiPs. b) Different region of the same sample as in (a) showing occurrence of thin fibrillar structures. c) EM image of POPC αSyn-LiPs, no fibrillar structure was detected in this or any other region of the sample as well as in repetition experiments. d) CD spectra of the POPC (red) and POPG (blue) αSyn-LiPs (same condition as used for the respective EM images). e,f) Zoom into selected regions in POPG αSyn-LiPs showing possible connections between αSyn-LiPs and fibrils.

**Fig. 4:**
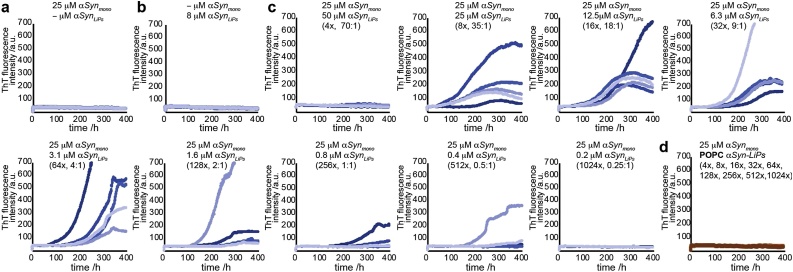
**aSyn-LiPs can induce primary nucleation**. a–d) ThT aggregation assays under quiescent conditions. Under these conditions neither aggregation of αSyn in the absence of αSyn-LiPs (a) nor of αSyn-LiPs on their own (b) is observed. Each plot contains data of five replications of the indicated condition. c) Variation of αSyn-LiP level in the presence of constant monomeric αSyn starting concentrations. In addition to concentration of monomeric αSyn, also the concentration of αSyn in LiPs is provided for each plot. Numbers in parentheses refer to estimated excess of monomeric αSyn over αSyn-LiP particles (assuming an average composition of 8 αSyn per LiP (Eichmann et al., 2016)). In addition, the respective molar ratios of lipids to added monomeric αSyn are given. d) All conditions as shown in (c) but using POPC αSyn-LiPs (all 45 curves are shown in one plot).

**Fig. 5:**
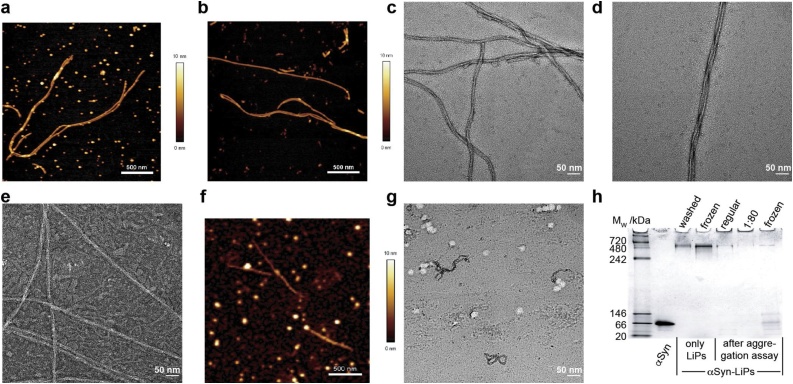
**Sample properties at the end of the aggregation assays.** AFM images of αSyn fibrils grown in the presence of POPG αSyn-LiPs, before centrifugation (a) and after removal of the supernatant (b). c + d) EM image of αSyn fibrils grown in the presence of POPG αSyn-LiPs before centrifugation (condition 64x in Fig. 4c). e) EM image of αSyn fibrillar structures grown in the presence of POPG αSyn-LiPs that did not lead to sizable ThT signal increase (condition 4x in Fig. 4c). f + g) AFM and EM image of POPG αSyn-LiPs control samples at the end of the aggregation assays (without addition of excess of monomeric αSyn, Fig. 4b). d) Native PAGE of monomeric αSyn as well as αSyn-LiPs in indicated conditions (washed refers to high-salt washed; frozen = non-washed, flash frozen and stored at −20 °C; regular = non-washed, non-frozen; 1:80 = preparation with high salt and adapted molar ratio of αSyn to lipids of 1:80). Note that amount of monomers added in aggregation assays is identical to amount loaded in the free αSyn control (first lane).

Samples at different time points were used for EM studies. Freshly prepared αSyn-LiPs (concentrated to 150 μM αSyn) were flash frozen in liquid nitrogen after SEC elution (used for Fig. 3). In addition, samples after the ThT quiescent aggregation assays (Fig. 4) were collected from the respective assay wells (used for Fig. 5). All samples were kept at the used phosphate buffer, reducing possible preparation artifacts but leading to larger background staining artifacts. Negative stained samples were prepared on plasma-cleaned formvar-carbon-coated copper grids with a 2% uranyl acetate stain solution. Electron microscopy images were taken on a CM20 microscope operated at 200 kV.

### 2.9 Circular dichroism (CD)

The secondary structure of αSyn-LiP assemblies was determined using a J-815 spectropolarimeter (JASCO, Tokyo, Japan). Samples of αSyn-LiP assemblies were prepared in (4 mM NaPi pH 7.4; 10 mM NaCl) with the working concentration of LiPs at 4 μM. For CD measurements, samples at 200 μl were loaded into a 1 mm path length quartz cuvette and spectra were recorded from 195 to 260 nm, using a scanning speed of 50 nm/min and a bandwidth of 2 nm, at 20 °C. The final spectrum of each sample was averaged based on 10 accumulations. The signal of the buffer was subtracted manually.

### 2.10 Native polyacrylamide gel electrophoresis (PAGE)

For gel casting 16%, 8%, and 4% acrylamide/bisacrylamide solutions (37.5:1, Carl Roth) were prepared in 250 mM Tris−HCl pH 7.4. Tetramethylethylendiamin (TEMED) and ammonium persulfate were added to a final concentration of 0.05% each. Afterwards, gels were immediately cast by layering the solutions over each other between two glass plates for polymerization with 16% acrylamide/bisacrylamide at the bottom (˜ 1.5 cm), 8% in the middle (˜ 4 cm), and 4% on top (˜ 1.5 cm). Samples were prepared by adding non-denaturing loading buffer (final concentrations 75 mM Tris−HCl pH 7.4, 10% glycerol, 0.02% bromophenol blue (w/v)). NativeMark™ Unstained Protein Marker (ThermoFisher Scientific) was loaded as a reference. The samples were separated by applying 25 mA per gel. Afterwards, gels were fixed in 10% ethanol, 3% phosphoric acid (v/v) for 15 min at room temperature and stained with colloidal Coomassie for at least one hour at room temperature (prepared from 0.02% Coomassie Brilliant Blue G-250 (w/v), 5% aluminium sulfate octadecahydrate (w/v), 3% phosphoric acid (v/v), and 10% ethanol (v/v)). Images were acquired using the ChemiDoc MP™ Imaging System (Bio-Rad).

### 2.11 Atomic force microscopy (AFM)

Samples were taken at the end of aggregation experiments, before or after centrifugation at 16,000∙*g* for 30 min. The pelleted fibrils were resuspended in the same volume of PBS (10 mM Phosphate Buffer pH 7.4, 137 mM NaCl, 3 mM KCl, 0.02% NaN_3_). Samples were diluted in PBS to a final concentration of 10 μM and applied onto freshly cleaved mica for 5 min. Excess salt was removed by gently rinsing sample with water and dried with a slow flow of N_2_. AFM images were taken in air, using a Nanowizard III atomic force microscope (JPK). Imaging was performed using tapping mode with a silicon cantilever with silicon tip (OMCL-AC160TS, Olympus) with a tip radius of 7 ± 2 nm and a force constant of 26 N/m.

## 3 Results and discussion

### 3.1 Preparation and initial characterization of αSyn-LiPs

Following previously described methods (Eichmann et al., 2016), we assembled stable nanoscale lipid particles using anionic 1-palmitoyl-2-oleoyl-sn-glycero-3-phospho-(1′-rac-glycerol) lipids (POPG) and αSyn as scaffold protein. αSyn has been shown to be N-terminally acetylated in cellular environments and its acetylation is thought to act as an important mode of regulation of protein-membrane association (Nemani et al., 2010; Dikiy and Eliezer, 2014). Therefore, in addition to non-acetylated αSyn, which was used in the initial studies of in vitro lipid particle formation (Eichmann et al., 2016, 2017), we also tested lipid particle formation using acetylated αSyn. The resulting αSyn lipoparticles (αSyn-LiPs) were characterized using size-exclusion chromatography (Fig. 1). The αSyn-LiP preparations with the two different αSyn variants show very similar SEC profiles (Fig. 1a) confirming that acetylated αSyn can also form αSyn-LiPs. Since this variant is physiological more relevant, in particular in the context of lipid interactions, only acetylated αSyn was used for the following experiments. In general, both SEC profiles are in line with previous results in which αSyn-LiPs elute close to the void volume of the used Superdex 200 columns (Eichmann et al., 2016, 2017).

In order to investigate the influence of storage capabilities conditions on αSyn-LiPs, we performed SEC experiments after storage at 4 °C for one day, one week, as well as after flash freezing with liquid nitrogen and storage at −20 °C. The similarity of the resulting SEC profiles (Fig. 1b) suggests that αSyn-LiPs can be stored for several days at 4 °C or can be frozen for storage, largely facilitating their handling and usage for various assays.

Notably, αSyn is known to interact with negatively charged membrane surfaces (Fusco et al., 2014; Rhoades et al., 2006; Jo et al., 2000; Bodner et al., 2009; Theillet et al., 2016). This interaction is not driven by the net negative global charge, but rather by the partial positive charge in the N-terminal part of the protein. Therefore, when assembling αSyn-LiPs using anionic lipids it is unlikely that αSyn will only stabilize the hydrophobic edges of the lipid bilayer in the same manner as the membrane scaffold protein in the nanodisc system, but may also bind to the membrane surface (and/or partially insert at various positions in the bilayer). In order to decrease the electrostatic contributions of a potential αSyn membrane surface interaction, we increased the ionic strength of the buffer by changing the NaCl concentration from 50 mM (low salt) to 300 mM (high salt). SEC analysis of αSyn-LiPs, initially prepared using low-salt buffer and then incubated in high-salt buffer, shows that high-salt concentration results in dissociation of αSyn monomers from αSyn-LiPs (Fig. 1c, red). When reinjecting high-salt washed αSyn-LiPs, no further αSyn monomers are detached, suggesting that the remaining particles are stabilized predominantly by hydrophobic interactions (data not shown). When αSyn-LiPs are directly prepared in high-salt conditions using the previously reported αSyn-to-lipid molar ratio of 1:40 during αSyn-LiPs assembly, a high fraction of monomeric αSyn is again visible in the SEC profile (Fig. 1c, orange). Interestingly washing with 300 mM NaCl or full preparation in 300 mM NaCl leads to a comparable amount of αSyn monomers present in the sample. This suggests that, when using anionic lipids, the used protein-to-lipid ratio may not be optimal to effectively form disc-shaped particles in which the amphipathic properties of αSyn-helices are exploited to stabilize the hydrophobic membrane edges. While at a molar ratio of 1:40 (αSyn:lipid) nearly all αSyn is incorporated into αSyn-LiPs at low-salt conditions (Fig. 1c, black), only about half of the total αSyn is incorporated when electrostatic interactions are weakened at the same protein:lipid ratio (Fig. 1c, orange, red). We therefore also tested high salt αSyn-LiP formation at ratios with 2-fold increased excess of lipids, i.e. a protein:lipid molar ratio of 1:80. The resulting SEC profile (Fig. 1d, green) shows a considerably decreased fraction of monomeric αSyn as compared to high salt prepared αSyn-LiPs at protein:lipid molar ratios of 1:40 (Fig. 1c, orange). This data is in line with the picture that under conditions which lower membrane surface attachment (e.g. high salt or possibly also usage of neutral lipids), αSyn-LiPs are formed with roughly 2-fold less αSyn molecules per LiP.

To experimentally determine the protein-to-lipid ratio, we carried out quantitative Fourier Transform Infrared (FTIR) spectroscopy (see methods and supplementary Fig. S1 for more details). The data show a protein-to-lipid ratio of 1:35 for αSyn-LiPs prepared at low-salt conditions. This value is very well in line with the previously estimated ratio of 1:40 (Eichmann et al., 2016). When using αSyn-LiPs prepared under high-salt conditions, the ratio changes considerably to 1:105 consistent with the picture that the low-salt αSyn-LiPs carry a substantial amount of αSyn attached to the lipid surface via electrostatic interactions. According to the FTIR data about 2/3 of the protein may be in such a conformation in the low-salt αSyn-LiPs.

We further characterized the hydrodynamic radius of the resulting particles using Dynamic Light Scattering (DLS). While the resulting overall size distribution is in line with the expected αSyn-LiPs properties, i.e. rather heterogenous particles with diameters from 10 to 44 nm (Fig. 1e), the sample heterogeneity renders accurate particle sizing via DLS, in particular when applying polydisperse data analysis, rather unreliable (see Fig. S2 for more details). This complicates detection of presumably small variations of particle sizes due to changing conditions as e.g. induced by attachment of monomers to preformed αSyn-LiPs. However, the DLS data are consistent with the particles sizes also seen in negative stained electron microscopy (EM) of the same samples (vide infra).

To more reliably detect smaller changes on the particle sizes, we used microfluidic diffusional sizing as an emerging alternative to DLS (Arosio et al., 2016), which is particularly well suited for particles in the size range of αSyn-LiPs. The principle of this type of measurement is that the diffusion of proteins and protein complexes in a laminar flow regime within a microfluidic channel is quantified. At the entrance of the channel, one half is filled with water and the other half with protein solution. Laminar flow ensures that no turbulent mixing occurs and the two fluid streams stably flow in parallel. The amount of protein that diffuses across the channel perpendicular to the flow direction is quantified by measuring the concentrations at the two symmetric channel outlets. Large particles, such as αSyn-LiPs, require a comparatively slow flow rate, in order to provide enough time for a significant amount of diffusion to occur. Protein quantification is based on a latent fluorophore (Yates et al., 2015) which reacts with the protein molecules after they have left the main channel, and which renders the proteins fluorescent.

Regularly-prepared (low salt + flash frozen) αSyn-LiPs display a hydrodynamic radius of 16.9 ± 0.6 nm (Fig. 1f). When measuring high-salt-washed αSyn-LiPs after SEC separation of monomeric αSyn, a hydrodynamic radius of 13.8 ± 0.4 nm is obtained, which most likely reflects the actual size of αSyn-LiPs without membrane surface-attached αSyn. Consistently, a comparable hydrodynamic radius of 12.7 ± 0.2 nm is detected for αSyn-LiPs assembled with a protein:lipid molar ratio of 1:80 in high-salt conditions. Note that the used SEC column (Superdex 200) displays a rather limited separation efficiency in this size regime, explaining why no size difference was detected in the respective SEC profiles.

We additionally tested whether monomeric αSyn, which shows a hydrodynamic radius of 2.9 ± 0.1 nm, attaches to high-salt-washed αSyn-LiPs (in low-salt conditions). Indeed, the addition of 16-fold molar excess of monomeric αSyn (16 αSyn monomers added per LiP) significantly increased the hydrodynamic radius of the particles to 22.1 ± 1.8 nm. Note that the microfluidic measurement yields the average hydrodynamic radius of all particles in the sample. The presence of larger amounts of free monomeric αSyn molecules would therefore lead to an apparent decrease in measured hydrodynamic radius of the sample. The measured value of 22.1 nm consequently suggests that a large fraction of the added αSyn monomers attaches to the αSyn-LiPs. Since the size of the “16-fold loaded” αSyn-LiPs is considerably larger than the size of the αSyn-LiPs formed at low ionic strength, the microfluidic diffusional sizing data also suggest that low salt αSyn-LiPs still have unoccupied binding sites for αSyn on the membrane surface.

In general, αSyn-LiPs which have not been in contact with a high ionic-strength solution are well-suited for further usage in different assays (vide infra), however one should keep in mind that these (low salt) αSyn-LiPs are formed with a higher number of αSyn per particle compared to those formed in, or washed with, higher ionic-strength buffer. While it is not fully clear where these additional αSyn molecules are located, binding to the lipid bilayer surface, i.e. interaction with the negatively-charged lipid head groups, would be one simple explanation consistent with the data obtained in this study. It should be highlighted that this also suggests that the estimation of the αSyn-LiP concentration based on measurements of the αSyn absorbance will be altered due to the different amounts of αSyn per particle at different ionic strengths. It is important to take this aspect into consideration, in particular for quantitative measurements of aggregation kinetics.

### 3.2 αSyn-LiPs in aggregation assays

Using a similar setup as reported for MSP1D1-derived nanodiscs (NDs) (Viennet et al., 2018), we explored the influence of αSyn-LiPs on αSyn amyloid-fibril formation. Initially we used a conventional GB-assay that reports on the effect of αSyn-LiPs on the lipid- independent αSyn aggregation pathway. To directly compare the results of αSyn-LiPs to the previously characterized effects of NDs (Viennet et al., 2018) we prepared NDs and αSyn-LiPs in parallel and performed a GB-assay simultaneously for both systems on the same 96-well plate (Fig. 2). Depending on the ratio of added αSyn monomers to either αSyn-LiPs or to NDs, both lipid systems can either inhibit aggregation or accelerate aggregation (Fig. 2a,b). Interestingly while a ratio of 16 added αSyn monomers per ND leads to an aggregation-accelerating behaviors (Fig. 2a, 16:1), the same ratio is in the inhibiting regime in the case of αSyn-LiPs (Fig. 2b, 16:1). This is well in line with the increased size of the αSyn-LiPs as compared to MSP1D1 NDs as well as the αSyn lipid-binding modes identified in our previous study (Viennet et al., 2018).

In general, anionic lipids in liposomes or in nanodiscs are capable of inducing primary nucleation of αSyn (Galvagnion et al., 2015; Viennet et al., 2018). To investigate whether anionic lipids in αSyn-LiPs also show nucleation-inducing properties, we investigated αSyn-LiPs with 100% POPG lipids via negative stain electron microscopy (EM). Surprisingly, the αSyn-LiPs already show directly after their SEC elution, in addition to the expected disc-like particles (Fig. 3a), the occurrence of thin fibrillar structures (Fig. 3b). While the amount of fibrils is difficult to quantify via EM, the polydisperse DLS data analysis (Fig. 1e) reports a fraction of (only) 0.03% very large particles (>500 nm, 0.03% volume weighted, 16% intensity weighted, also see supplementary information Fig. S2). Since the SEC elution peak itself appears directly after the void volume of the used column, the respective samples were flash frozen in liquid nitrogen directly after SEC elution, and EM data was directly recorded after a short thawing step, it is likely that the fibrilar strucutures already formed during αSyn-LiP assembly, which is a rather slow process, e.g. due to the prolonged incubation with Biobeads for detergent removal. To investigate whether the fibrillar strcutures are induced by the anionic lipids or are just an artifact of the αSyn-LiP assembly process itself, we also prepared αSyn-LiPs containing 100% 1-palmitoyl-2-oleoyl-glycero-3-phophocholoine (POPC) lipids. The POPC αSyn-LiPs were prepared in parallel to POPG αSyn-LiPs. EM data recorded afer SEC elution (supplementary information Fig. 3) do not show any fibrillar structures for POPC αSyn-LiPs in two different samples and over 20 different scan regions (Fig. 3c provides one example).

We also characterized the respective POPG and POPC αSyn-LiPs via circular dichroism (CD) spectroscopy (Fig. 3d). The resulting CD spectra of the two samples are very similar and in line with the expected secondary structure, i.e. an amphipathic α-helix for first approx. 100 residues and random coil conformations for the remaining C-terminal residues, as seen in NMR spectra of comparable αSyn-LiPs (Eichmann et al., 2016). Note that the remaining small deviation between the two CD spectra would also be in line with a very small population of β-sheet rich fibrils in the POPG αSyn-LiPs sample.

Overall, our data suggest that POPG αSyn-LiPs after SEC elution already contain a small fraction of fibrillar structures, which were induced by the presence of anionic lipids. Having a closer look at the fibrillar structures in the EM images shows that to some extend the fibrils colocalize with αSyn-LiPs (Fig. 3e,f). In general, it cannot be fully excluded at this point that αSyn-LiPs and fibrils cluster during the drying process on the EM grid. However, such a clustering should in principle result in αSyn-LiPs appearing at random positions on the fibrils. While such connections are also observed, a rather large fraction of fibrils appear to ‘grow out’ of the LiPs. Such defined start/end points may indeed suggest that the EM images captured early stages of lipid induced αSyn aggregation.

Interestingly, it has been shown that aSyn can also reshape lipid vesicles into lipid nanotubes consisting of either a monolayer of lipids (micellar tubes) or of a lipid bilayer in a cylindrical arrangement (Mizuno et al., 2012). The observed fibrillar structures also share some similarities with these micellar lipid tubes. While it is difficult to distinguish small protein fibrils from lipid tubes in the negative stained EM images, amyloid-fibril-specific ThT fluorescence increase can be used to distinguish between the two species, once sufficient fibrils are formed. In order to test whether nucleation-inducing properties of POPG αSyn-LiPs can be monitored via ThT aggregation assays, we performed additional ThT assays under conditions that do not promote the formation of detectable quantities of amyloid fibrils in the absence of LiPs. Such a setup is provided by using quiescent assay conditions (no glass bleads, no shaking) (Galvagnion et al., 2015). The absence of a glass bead facilitates usage of smaller sample volumes. We therefore carried out the assay using a volume of 30 μl per well in a 384-well plate format. In general, we observed that this assay has limitations in reproducibility and shows variations in the ThT profiles of wells with the same conditions in particular in respect to total ThT fluorescence intensity. In general, reproducibility in αSyn aggregation assays is a well-known challenge (Wordehoff and Hoyer, 2018). It is therefore not unexpected that the rather slow kinetics observed in the αSyn-LiP nucleation assay also propagates detectable differences in wells replicating the same conditions. However, the assay format also facilitates usage of a higher number of replications for each condition. We therefore carried out 5 replications for each condition and the resulting ThT profiles show a clear trend, despite their intrinsic variation. Our data show that (i) αSyn does not aggregate in the absence of αSyn-LiPs (Fig. 4a), (ii) αSyn-LiPs on their own do not form ThT-detectable amyloid fibrils (Fig. 4b), (iii) αSyn-LiPs can induce amyloid fibril formation at specific ratios of αSyn to αSyn-LiPs (Fig. 4c), and (iv) αSyn-LiPs formed with neutral POPC lipids do not induce aggregation at any of the tested αSyn to αSyn-LiPs ratios (Fig. 4d).

Note that αSyn-LiPs showing fibrillar structures were used as starting material for the aggregation assays (Fig. 4b,c). In case the fibrillar structures represent protein fibrils and not lipid tubes, it would be likely that the fibrils can act as seeds in the aggregation assay. The shape of the resulting kinetic profile however still shows a long lag phase, indicative of primary nucleation events, in all conditions resulting in ThT-detectable fibrils. The time frame of the corresponding lag phase is also considerably larger than the sample preparation time before the assays, suggesting that a potential seeding effect originating from the pre-existing fibrillar structures is rather small. Considering that all negative controls consistently do not show any ThT increase, it can be stated that αSyn-LiPs containing anionic lipids induce primary nucleation.

Interestingly, ThT assays carried out using SUV preparations with comparable lipid composition show induction of primary nucleation at comparable lipid:αSyn (monomer) ratios (Galvagnion et al., 2015). However, SUVs did not induce detectable aggregation at the used NaCl concentration of 50 mM, even at higher αSyn concentration (Galvagnion et al., 2015). This observation suggests that αSyn-LiPs show similar properties as SUV and may be even more potent in inducing primary nucleation than SUVs. However, a more thorough mechanistic analysis beyond the scope of this work will be required to quantify the kinetic rate constants as well as the molecular determinants of αSyn-LiPs-modulated αSyn aggregation. While the contributions of potential nucleation events prior to the start of the assay may or may not complicate data analysis, our data demonstrate that αSyn-LiPs provide an interesting tool in the investigation of lipid-induced αSyn aggregation.

To obtain insights into the sample properties at the end points of the aggregation assays, we recorded AFM and EM images of selected samples. Our data show that extended fibrils with a morphology comparable to αSyn fibrils obtained in regular GB-assays have been formed in the presence of αSyn-LiPs (Fig. 4a–d). Note that the samples were obtained in conditions in which the αSyn-LiPs induced fibril formation (Fig. 4c, 128x for AFM, 64x for EM). αSyn fibrils grown in the presence of SUVs show a clearly distinct morphology when the plateau of ThT fluorescence is reached, since their growth is strongly affected due to the SUV lipids (Galvagnion et al., 2015). These SUV-induced fibrils appear to be kinetically trapped, as it was recently found that an increase in temperature is able to induce their conversion into mature fibrils (Brown et al., 2018). Interestingly, when directly imaging the sample containing αSyn fibrils that were induced by αSyn-LiPs after the aggregation assay, a number of particles consistent in size and overall appearance with αSyn-LiPs are found (Fig. 5a). After centrifugation and removal of the supernatant the occurrence of these particles in the AFM image is largely reduced (Fig. 5b). While other contributions, such as drying-induced assemblies of monomeric αSyn cannot be fully excluded, this observation is in line with the presence of soluble αSyn-LiPs after the aggregation assay. EM images also show disc-like particles attached to mature fibrils (Fig. 5c). This could either be residual fibril αSyn-LiPs complexes as observed at the beginning of the aggregation assays (Fig. 3e,f) or again drying-induced clustering of mature fibrils and soluble αSyn-LiPs or amorphous aggregates. In any case, most fibrils are free of disc-like particles and show characteristic features (branching and twists) of mature αSyn amyloid fibrils (Fig. 5c,d).

Surprisingly, EM images of αSyn monomers incubated with the highest amount of POPG αSyn-LiPs (Fig. 4c, x) also show clear fibrillar structures (Fig. 5e), despite showing no increase in ThT signal (Fig. 4c, x). As compared to the EM data of the other conditions, this sample displays larger heterogeneity (areas with and areas without fibrillar structures, not shown). In addition, the EM image suggests that the fibrillar structures are surrounded by a rather large number of heterogenous particles, possibly LiPs (Fig. 5e). It is at this point not clear whether the total amount of fibrils in this sample is too low to induce a detectable ThT signal increase, or whether the fibrillar structures are lipid (bilayer) tubes, or whether a different fibril morphology and/or lipid coating weakens ThT interactions.

The AFM and EM images of the αSyn-LiPs control sample at the end of the aggregation assays (i.e. αSyn-LiPs without addition of αSyn monomers) still show particles most likely reflecting intact αSyn-LiPs (Fig. 5f,g) and low amounts of short fibrillar structures as already present at the beginning of the assay (Fig. 3).

To detect remaining monomeric αSyn as well as intact αSyn-LiPs, we additionally carried out a native PAGE analysis of selected samples after the aggregation assays. The resulting gel shows clear bands for the monomeric αSyn reference as well as for freshly prepared αSyn-LiPs (Fig. 5h). The latter appears between the molecular weight markers for 480–720 kDa. A comparable band is also observed for frozen αSyn-LiPs. Notably, also weak bands at this position are observed after the aggregation assay, supporting the view that a fraction of αSyn-LiPs are still intact at the end points of the aggregation assay. A rough estimation based on the band intensity and the loaded αSyn-LiPs amount, however suggests that the fraction of intact αSyn-LiPs is rather low (<10%). Interestingly, the amount of monomeric αSyn loaded onto the gel for the reference sample (first lane) reflects the amount added at the beginning of the respective aggregation assay. Since no or only very weak bands are observed for monomeric αSyn after the aggregation assay, our data show that most monomeric αSyn molecules have either been incorporated into αSyn-LiPs and/or have formed larger aggregates. The rather weak bands for αSyn-LiPs are in favor for the latter, suggesting that αSyn aggregation was very effective in the presence of αSyn-LiPs.

## 4 Conclusion

Overall, we have shown that αSyn-LiPs can be used to induce, accelerate or inhibit αSyn amyloid fibril formation. While our results are well consistent with a planar lipid bilayer stabilized by surrounding αSyn molecules with an approx. 4-to-8-fold increased surface area as compared to MSP1D1 nanodiscs, it should be pointed out that we cannot exclude different molecular arrangements of αSyn and lipids. In addition, we have shown that usage of anionic lipids in combination with low ionic strength of the sample buffer leads to αSyn-LiPs formed with a higher number of αSyn molecules per LiP. Since about half of the αSyn proteins can be detached from these αSyn-LiPs by increasing the ionic strength of the buffer while the other half remains attached to LiPs, we attribute this observation to the contribution of an electrostatically driven binding of αSyn to the negatively charged membrane surface.

The presence of αSyn-LiPs in the used aggregation assays evidently induces distinguishable modulations of the aggregation behavior. Our EM data show connections between short fibrillar structures and αSyn-LiPs that could reflect on early lipid induced nucleation events. However, more thorough investigations will be needed to understand the formation and role of these fibril-LiP complexes and whether they play a role in the amyloid fibril formation process. While very consistently only the presence of αSyn-LiPs with anionic lipids led to ThT detectable αSyn fibrils in quiescence aggregation assays, we also observed limitations in well-to-well reproducibility. In general, reproducibility is a common problem in αSyn aggregation assays and it is at this point not clear whether αSyn-LiPs are prone to induce variations in the aggregation assays or whether assay conditions can be further optimized to increase reproducibility. Nevertheless, our data clearly demonstrate that αSyn-LiPs display useful features including (i) a very strong capability to induce primary nucleation, (ii) the possibility to store frozen αSyn-LiP stock solutions, simplifying handling and minimizing artifacts by batch-to-batch variations, and (iii) a not detectable influence on the morphology of fibrils that have formed and grown in the presence αSyn-LiPs. We therefore anticipate that αSyn-LiPs offer an attractive tool, complimentary to other setups, to study various processes of αSyn amyloid fibril formation.

## Competing interests

The authors declare no competing interests.
