# Supplementary material for: α-Synuclein-derived lipoparticles in the study of α-Synuclein amyloid fibril formation
Source: Chem Phys Lipids. 2019 May;220:57–65. doi: 10.1016/j.chemphyslip.2019.02.009 (PMC6451039; doi:10.1016/j.chemphyslip.2019.02.009)
Supplement: Supplementary file 1 [file mmc1.pdf]

Supporting Information

α-Synuclein-derived lipoparticles in the study of α-Synuclein amyloid fibril formation

Marcel Falke[1], Julian Victor[1], Michael M. Wördehoff[1], Alessia Peduzzo[1], Tao Zhang[1], Gunnar F. Schröder[2], Alexander K. Buell[1], Wolfgang Hoyer[1], Manuel Etzkorn[1,2*]

[1]Institut für Physikalische Biologie, Heinrich-Heine-University Düsseldorf, Germany

[2]Institute of Complex Systems (ICS-6), Forschungszentrum Jülich, Germany

Supporting Figures:

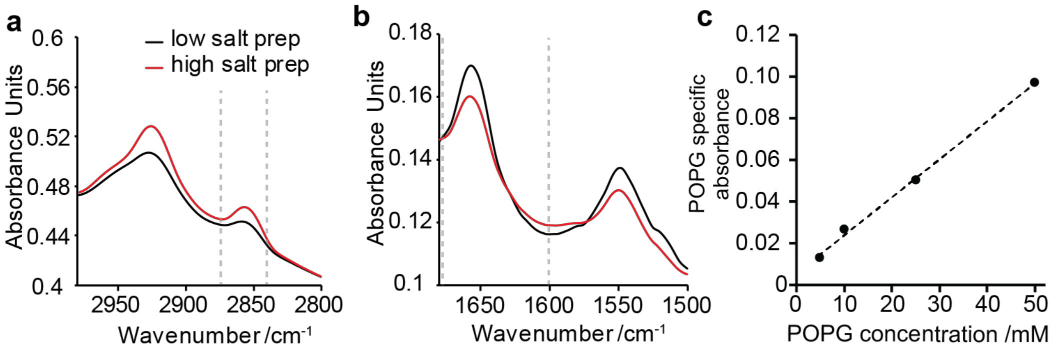

Figure S1. ***FTIR data of αSyn-LiPs after SEC***. *Spectral regions used for quantification of lipid (a) and protein (b) amounts. Baseline adjusted spectra of αSyn-LiPs prepared in low- (black) or high-salt (red) conditions are shown. While protein quantification is done automatically (using multiple spectral regions), lipid quantification requires a lipid-specific calibration curve, which we recorded using different concentrations of POPG in constant concentration of Na-cholate (c). Final lipid quantification was carried out after subtracting the respective buffer background spectra in triplicate experiments.*

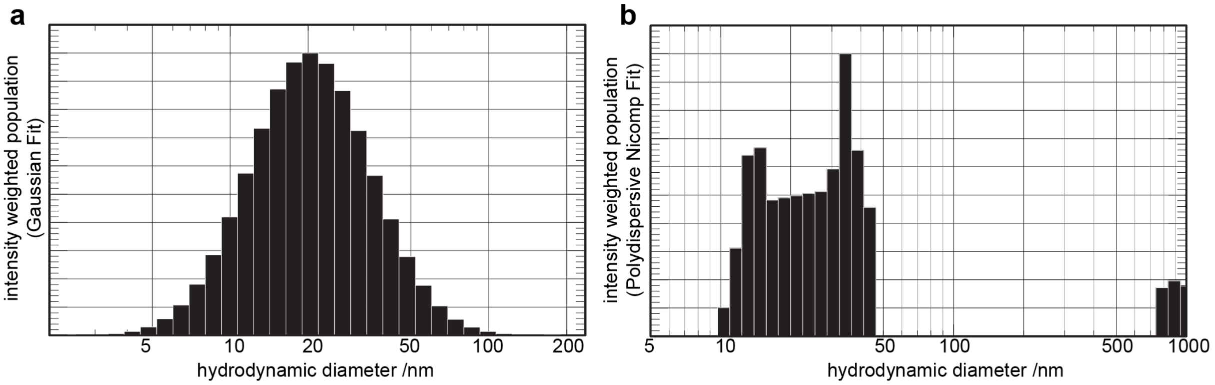

Figure S2. ***DLS data of POPG αSyn-LiPs after SEC***. *Histograms are based on the identical measurement as shown in Fig. 1e, but using different fitting algorithms. a) Result of intensity-weighted gaussian fit, assuming a monodisperse particle distribution. The average particle size obtained with this method is similar to the one obtained using microfluidic sizing, which also reports on the average value of all particles (Fig. 1f). b) Results of intensity weighted polydispersive fit using the instrument's internal, so called Nicomp, algorithm. The histogram again shows heterogenous particle distribution in the range of 10-50 nm. In addition a fraction of large particles (> 500 nm) with an overall intensity contribution of 16 % is detected. Since the DLS measurements were recorded on the same sample, which also shows fibrillar structures in EM images (Fig. 3b,e,f), it can be assumed that these structures contribute to the large particles sizes in the DLS histogram. Note that the intensity-weighted distribution is shown, which, due to the large intensity dependence of the DLS signal, translates to a population of below 0.1 % fibrillar structures (volume weighted).*

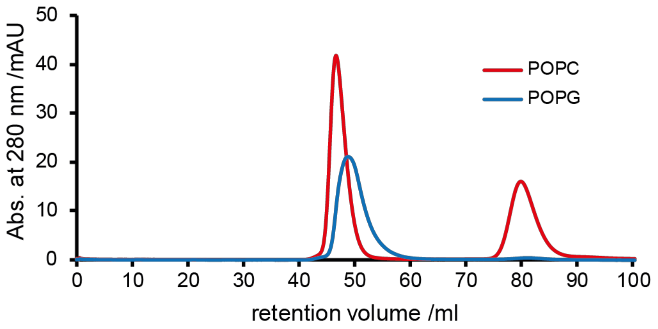

Figure S3. *Preparative SEC data of freshly prepared αSyn-LiPs assembled with POPC (red) or POPG (blue) lipids. Both preparations were carried out in parallel and under identical (low salt) conditions. In line with results of high-salt treatment of POPG αSyn-LiPs (Fig. 1c), the usage of the neutral POPC lipids also results in larger fraction of monomeric αSyn not attached to the LiPs at the used protein-to-lipid ratio of 1:40.*
